# Supplementary material for: Leprosy as immune reconstitution inflammatory syndrome in patients living with HIV: Description of French Guiana’s cases over 20 years and systematic review of the literature
Source: PLoS Negl Trop Dis. 2022 Mar 4;16(3):e0010239. doi: 10.1371/journal.pntd.0010239 (PMC8947598; doi:10.1371/journal.pntd.0010239)
Supplement: Attachment — Submitted filename: Response letter.docx [file pntd.0010239.s003.docx]

**RESPONSE LETTER**

Dear reviewers,

Thanks for reviewing our manuscript and for your remarks. We have made significant changes to our manuscript following your advice. Please find here our step-by-step response to your comments about "Leprosy as immune reconstitution inflammatory syndrome in patients living with HIV: 20 years of French Guiana experience and systematic review" for consideration at PLOS Neglected Tropical Diseases.

Sincerely,

Alice Mouchard

Corresponding author

**Response to reviewer 1**

“The study objectives are not clearly articulated.”

Thank you for your pertinent remark. We have reworded the last paragraph of the introduction to state more clearly the objectives and organisation of the article (line number 107-111).

“The population has been described. No statistical tests have been carried out just reporting of descriptive statistics”
In view of the relatively small amount of data, we did not consider it relevant to carry out statistical tests apart from the description by position criteria (averages and medians) and dispersion criteria (minimum-maximum) when we considered that this information was meaningful. We reported missing data because they were frequent.

“The tables are too busy and can be edited just to present relevant data.”

We performed a simplification of tables and proposed the full description as appendix tables to the journal.

“My biggest concern is after 20 years of exhaustive literature search among the n =246, only 22 had HIV co-infection and only 6 people could be found to have leprosy-IRIS? That suggests that it is not as much a healthcare problem as the authors indicate in the introduction to build their argument for the study”

Thanks for your remark: in fact, the healthcare problem in French Guiana is leprosy (aka more than 1 cases/10 000 inhabitants). It is true that leprosy as IRIS among PLHIV is not a healthcare problem due to its much lower incidence as described underneath. According to our figures in French Guiana, among the 246 leprosy patients followed up between 2000 and 2020, about 8% (22/246) were also co-infected with HIV. In this group of co-infected patients, about 27% (6/22) developed leprosy in IRIS, so this phenomenon is not uncommon. Leprosy and HIV are major public health problems worldwide, and we do not claim in our article that leprosy as IRIS is one of them: our aim was to draw the attention of clinicians to this entity and to describe it.

“The authors mention that leprosy and HIV seem to be reported to progress independently, but they have evidence that due to access to retroviral therapy, that’s not the case, which might be true but 6 individuals in 20 years doesn’t inspire confidence for that argument.”

Studies in the 1990s suggested that HIV infection and leprosy evolved on their own, but these studies took place before the introduction of antiretrovirals.

“The manuscript is a combination of case-reports and systematic review, and the title needs to reflect that”

We have changed the title so that it more accurately describes the content of the article: “Leprosy as immune reconstitution inflammatory syndrome in patients living with HIV: description of French Guiana’s cases over 20 years and systematic review of the literature”.

“The manuscript needs to be shortened to drive home a succinct point, and as it is more of that perhaps the cases were underreported or underdiagnosed and a call for attention on that subject, the conclusions should reflect that”
The manuscript was shortened (in material and methods as well as in the results and discussion), from 4624 to 3080 word (from Introduction to Conclusion).

The underreported/diagnosed issues have been reminded in the results and discussion as a major health issue.

“The spelling of French Guiana needs to be consistent”

Thank you for noticing that mistakes. I have standardized the spelling throughout the text. Despite what dictionaries and spellcheckers suggest, French Guiana is spelt with an I and not a Y according to the international denomination.

“The authors should include a line number when submitting manuscripts helps reviewers to pinpoint the changes”

Thanks for the advice, I have inserted the line numbers as you suggested.

“Discussion needs to be shortened a lot to include only major point of which there is a few”

Thanks for your remark. As explained above, we have shortened a lot the article to fulfill your reviews and to make a straight point. We hope that version will be more convenient.

**Response to reviewer 2**

“The paper is well design and adhere to the necessary guideline for the systematic review. there are some points needed to be added. Please see general comments”

Thanks for the comment, we have added the PRISMA 2020 checklist to justify that we have followed the guidelines for the systematic review

“Leprosy as immune reconstitution inflammatory syndrome in patients living with HIV: 20 years of French Guiana experience and systematic review. The paper is important and combine clinical and laboratory data to better describe the IRIS phenomena that triggers leprosy”

Thank you for your comment, as you understand, we wanted to better describe this IRIS phenomenon by compiling our Guianese cases and those reported scattered in several journals including a number published in the grey literature.

“There is some information that could be useful. For either the retrospective study or the systematic review, could authors retrieve whether patients are contact (social or household) that could be added”.

Indeed, contact information would be interesting. However, we did not have this information in the patient files in French Guiana. A majority of leprosy patients in French Guiana are Brazilians, who remain evasive when questioned. These people are often gold diggers in remote illegal camps in the forest, which makes it difficult to collect data during the interview. Futhermore, in the articles selected for the systematic review, the authors never described the absence or presence of contact with a leprosy person.

“Also, if authors could have number of CD4+ on the onset of IRIS/reactional leprosy as compared to IRIS/unreactional leprosy, that could help understand the pathogenesis of IRIS and more importantly leprosy. In this regard, these groups have different time of onset for IRIS/reaction or IRIS/leprosy per se?”

Thank you very much for raising this very interesting point.

I have calculated that the median time to onset of “IRIS/reactional leprosy” is **8** weeks (*i.e* IRIS + T1R or T2R at diagnosis of leprosy, indexed in table 2 by ^1^ (n = 37)). For “IRIS/unreactional leprosy” (i.e IRIS without T1R or T2R at any moment), the median time to onset was **10** weeks.

The median fold increase of CD4 in the “IRIS reactional leprosy” group was **3.2** whereas it was **2.5** in the “IRIS /unreactional leprosy “group.

### Thus, this additional analysis reinforces the impression that a high increase in CD4 count is involved in the rapid onset of reactional state associated with the development of leprosy. I have added a sentence to this effect in the renamed paragraph “Evidence of immune restoration and timing of onset” (line number 234-237)

**To be more precise, I did not include in this “IRIS/reactional leprosy” category IRIS with a leprosy reaction secondary to the introduction of MDT (indexed in table 2 by ^2^), nor those whose leprosy reaction chronology was not specified (for example patients described by Menezes et al. 2009)*

“For a broader readership IRIS types should be briefly introduced in the methods”

Unless I did not understand your question, I had defined the different types of IRIS in the Material and Methods of the previous manuscript, paragraph “Classification of leprosy as IRIS”. Nonetheless, that detailed definitions have been shortened on this resubmission to make this article easier to read.

“English can be improved”

We had the revised version of the manuscript proofread by one of our bilingual authors, hoping that these changes will suit you.

“In the last paragraph of the introduction “updated systemic review” should be updated systematic review, right?”

Indeed, thank you for noticing this typing error which I have corrected (line number 111)

“Please include reference of Deps and Lockwood: “We searched the files of all patients followed for leprosy in the dermatology department and extracted all files of HIV infected patients meeting the criteria defined by Deps and Lockwood.”

I have included the reference as per your advice (line number 123)

“In the table 2 the number of weeks in two patients are “16?”. What does it mean?”

Indeed, the number "16" followed by a question mark is not very clear. The article by Pereira et al, 2004, reports IRIS leprosy cases occurring between 8 and 24 weeks. I have averaged these two times as an estimate, which gives 16. I have changed the table to "8-24”.

“Authors should consider discuss the onset of IRIS for other infectious diseases such as tuberculosis. Similarities and differences should be pointed out”

Thank you for your interesting comment. We have discussed the similarity of the timeframe between the tuberculosis IRIS and the leprosy IRIS. However, we did not go further in terms of similarities and differences, because of the difference in pathophysiology between these two mycobacteria. We also chose not to discuss the other infectious IRIS. (line number 275-276)

“In the discussion the text: “IRIS is probably related to an early restoration of memory T-cell activity leading to an excessive inflammatory response in the presence of a latent infectious agent [61]. Despite the fact that (…)” needs better formatting”

Thank you for noticing this typo (I had mistakenly put the "underline" option on both sides of this sentence). We have also reworded the original sentence (line number 306-307)
